# Activation of the Large-Conductance, Voltage, and Ca^2+^- Activated K^+^ (BK) Channel in Acute Spinal Cord Injury in the Wistar Rat Is Neuroprotective

**DOI:** 10.3389/fneur.2018.01107

**Published:** 2018-12-18

**Authors:** Marianne Jacobsen, Kristen Lett, John Mark Barden, Gavin L. Simpson, Josef Buttigieg

**Affiliations:** Department of Biology, University of Regina, Regina, SK, Canada

**Keywords:** spinal cord injury, excitotoxicity, motor function, electrophysiology, immunohistochemistry

## Abstract

**Context/Objectives:** Spinal cord injury (SCI) results in significant neuronal and glial cell death resulting in impaired neurological and motor function. Uncontrolled Ca^2+^ entry results in excitotoxicity and cell death. In this study, we examine the use of a BK channel activator, Isopimaric acid (ISO), as a neuroprotective agent post-SCI as this channel is involved in regulating Ca^2+^ entry.

**Design:**By using a 25-g clip compression at the T6 level, we generated a SCI event in wistar rats. At 1 h post-injury we administered ISO (BK channel activator), the BK channel inhibitor iberiotoxin (IbTx), or a vehicle control for 4 weeks via mini osmotic pump (pump capacity). For 8 weeks post-injury, gait analysis of motor function was performed. At the end of 8 weeks, the extent of myelination in the spinal cord was assessed in addition to the electrophysiological profile.

**Results:**Our immunohistological data suggests that ISO treatment leads to an increase or preservation of myelinated axonal tracts. This was further supported by our electrophysiological studies which demonstrate higher compound action potential amplitudes and speed of transmission in ISO-treated animals compared to inj-non-treated. Finally, treatment with ISO significantly improved motor function in our test model.

**Conclusion:** In conclusion, activation of the BK channel during acute SCI may be a novel therapeutic target for acute SCI.

## Introduction

In north America there are over 1.3 million individuals with spinal cord injury (SCI) and an annual incidence rate of 30–70 cases per million people (1, 2). Mitigation of injury severity as well as return of normal function are primary concerns, however, current therapies are unable to do either. During SCI, there are typically two injury events. Primary damage is caused by the mechanical impact to the spinal cord from deformities of surrounding anatomical structures. Within minutes, the cellular membranes are disrupted, the myelin sheath starts to break down, and localized hemorrhaging of the spinal cord occurs. The secondary injury consists of uncontrolled cell depolarization, significant increases in reactive oxygen species (ROS) formation, inflammation and further cell death. The propagation of cell loss further exacerbates the severity of the injury (3, 4). There is significant loss of axonal tracts, with some surviving in the subpial rim of the spinal cord (5), however these tracts are often dysmyelinated or demyelinated resulting in loss of axonal function (6).

Under normal physiology, voltage-gated K^+^ (Kv) channels play a key role in repolarizing the membrane potential and reducing Ca^2+^ influx. While several Kv channels have been implicated in normal physiology and pathophysiology of the spinal cord (7–9), the role of the large-conductance, voltage and Ca^2+^- activated K^+^ (BK or Maxi K^+^) channel remains unknown. The BK channel, while closed at resting membrane potential, increases K^+^ conductance upon elevation of membrane voltage and Ca^2+^ influx (10–12). Thus, these ion channels act to reduce excitotoxicity and are a possible pharmacological target in the treatment of acute SCI (13).

We have previously demonstrated that BK channels normally reside in the juxtaparanodal region and are exposed after chronic SCI, and thus play a key role in the altered physiology of the chronically injured spinal cord (13). Our previous work demonstrated that inhibition of the BK channel on injured spinal cords with iberiotoxin (IbTx) leads to recovery of action potential amplitude as well as a prolonged action potential decay phase during chronic SCI (13). Thus, demonstrating that myelin loss during the acute phase exposes these ion channels, and in the chronically injured spinal cord, contributes to altered physiological function. A drawback of this study, was that it solely examined the physiology of the chronically injured spinal cord. While we did demonstrate the presence of this ion channel, we did not examine whether molecular manipulation of its activity during the acute phase of injury had any effect on functional outcomes (13). In this present study, we seek to address this gap in our understanding of the role of the BK channel during SCI.

While there are several isoforms of the BK channel, the predominant one expressed in the spinal cord are those from the stress regulated exon (STREX) (14). This subtype of ion channel is acutely sensitive to elevated ROS levels, as observed during acute SCI, and inhibit BK channel conductance (15). Here we questioned whether activation of the BK channel reduces severity of injury during the acute stage of SCI. We assess both physiological and motor outcomes in a rat model of SCI using immunohistochemistry, electrophysiology, and kinematics.

## Materials and Methods

### Animals

Adult female Wistar rats (RRID:RGD_11040548) were obtained from Charles River weighting approximately 300 g. Experimental protocols were approved by the local President's Committee on Animal Care of the University of Regina (Aup#14-11), in accordance with policies set up in the Guide to the Care and Use of Experimental Animals by the Canadian Council of Animal Care. Rats were housed in standard rat cages in a room maintained at 22 ± 2°C with a 12/12 h light/dark cycle with water and food provided ad lib. For all treatment groups, we selected the minimum amount of animals per treatment group that would allow us to develop statistically meaningful data. This is in accordance with University of Regina Animal Care guidelines.

### Injury Model

Rats (300 g) were anesthetized with 2% isoflourane and a 1:1 mixture with O_2_. Rats were given a T5-7 laminectomy, followed by a 25 g aneurism clip compression injury for 1 min. A micro aneurysm clip was purchased from Harvard Apparatus and was rated as 25 g of force. Calibration with a micro dynamometer was performed and the spring was determined to give a force of 0.246 g + 0.17 Newtons of force. Clip spring force was measured after every 5–10 surgeries. Injury of animals was found to be uniform after the procedure with similar range of motion. The injury site was covered with spongistan to protect the exposed tissue and to give support for the inserted catheter (see below). Animals were assigned to four different treatment groups: (1) BK channel activator, isopimaric acid (ISO, 20 mg/Kg; *n* = 12); (2), BK channel blocker iberiotoxin (IbTx, 0.1 mg/Kg; *n* = 11); (3) vehicle control [Injured-non-treated (Inj-No-Tx); *n* = 8]; and (4) laminectomy only (Lam; *n* = 3). Drugs were administered 1 h post-clip compression injury through a mini osmotic pump (Alznet, 4 weeks delivery). The mini osmotic pump was primed 24 h prior to surgery, to ensure that compounds were flowing at time of insertion. Briefly, the catheter was backfilled with the compound and the pump was filled. The catheter and pump were then stored in PBS overnight. During surgery, the next day, the pump was placed subcutaneously, and the catheter was secured to the musculature using sutures. Using a needle probe, a small incision was made through the meninges and the distal end of the catheter was inserted subdurally. Laminectomy animals also had a catheter inserted, but was not affixed to a pump. The pump administers a continuous stream of the compound contained in the pump at the set concentrations listed above for 4 weeks after insertion.

The bladders of rats that had received a SCI were expressed 3 times daily until reflexive bladder control had returned. Gait analysis, electrophysiological and immunohistochemistry experiments were performed on rats 8 weeks post-SCI. Individuals conducting the experiments were blinded to the treatments received by the animals.

### Immunohistochemistry

Animals were euthanized with an overdose of sodium pentobarbital (150 mg/Kg). After exsanguination, spinal cords were acutely isolated and fixed. Spinal cords that were to undergo immunohistochemistry were fixed via perfusion in 4% paraformaldehyde (PFA) in 0.1 M phosphate buffered saline (PBS), pH 7.4, and kept at 4°C. The cords were post-fixed in the same PFA-PBS solution with 10% sucrose overnight at 4°C, followed by incubation in 20%, and a final 30%sucrose solution in PBS for 24–48 h at 4°C for cryoprotection. Tissue was embedded in tissue freezing medium (TBS) on dry ice, and sagittal sections (20 μm) were mounted onto glass slides. Sections were dried for 10 min at room temperature, followed by a PBS wash for 10 min. Sections were blocked for 20 min in blocking solution consisting of 1x PBS containing 1% bovine serum albumin (BSA), 0.1% Triton X-100, and 0.3 M glycine, and incubated with primary antibody in blocking solution overnight at 4°C. Sections were dual stained with mouse anti-neurofilament (1:100; Novus Biologicals Cat# NB300-134 RRID:AB_10000761) which identifies the heavy neurofilament in axonal tracts and rabbit anti-myelin basic protein (MPB; 1:200; Abcam Cat# ab40390 RRID:AB_1141521) which identifies myelin. Slides were washed three times for 5 min with PBS, followed by incubation with secondary antibodies donkey anti-mouse Alexa Fluor 594 (1:500; Abcam Cat#ab150108) and donkey anti-rabbit Alexa Fluor 488 (1:1,000; Abcam Cat# ab150061 RRID:AB_2571722) for 1 h RT. Secondary antibody was washed off with PBS three times for 5 min, and 4, 6- diamidino-2-phenylindole (DAPI) and Flurosheild (GeneTex) added to the slides for counterstaining of the cellular nucleus. Slides were covered with a cover slip, and kept at RT. A Zeiss Axio Oberver Z1 epifluorescent microscope with Colibri illumination system and software were used for imaging sections and analysis. Antibodies were tested for specificity using known positive samples (e.g., MBP or NF positive samples) and optimized on these protein samples for optimal specificity and image intensity. Further controls utilized were omission of primary antibody to test for the specificity of the secondary antibody.

### Electrophysiological Recordings of Compound Action Potential From the Spinal Cord

In order to determine the functional capability of the spinal cord, electrophysiological studies were conducted to measure the electrophysiological profile of the spinal cord in treated and non-treated rats, similar to previous studies (13). After anesthetization of the animal with an over dose of sodium pentobarbital (150 mg/Kg), the spinal cords were removed from the animals and placed in ice cold PBS. The spinal cord then was dissected further. This included removal of the meninges and dissection of the cord into ~5 cm lengths. The entire procedure from spinal cord removal to dissection took place in oxygenated ice-cold artificial spinal cord fluid (ASCF, see below). These longitudinal sections of the spinal cord were placed in the recording chamber (see **Figure 2**) in room temperature ASCF (~25°C). Sections that were not being recorded were stored in oxygenated ice-cold ASCF. The recording chamber consisted of bipolar electrodes made of stainless steel wires (outside diameter 0.4 mm). There were 5 additional insulated supports to allow the spinal cord segment to rest in the chamber. The rostral parenchymal end of the spinal cord was placed in contact with the stimulating electrodes while the caudal end of the spinal cord was supported on insulated supports (**Figure 2**). A recording electrode was made from borosilicate glass to form a suction electrode. The electrode was placed at the caudal end. Suction was applied to ensure a good seal. The distance between the stimulating electrode and the recording electrode was measured. The distance between the stimulating and recording ends is essential to achieving a high signal to noise ratio of recordings. To ensure electrical isolation and avoid shunting effects, the suctioned end was lifted above the solution. The recording electrode was connected to the head stage of a molecular devices 200B amplifier (Axon instruments). During all recordings, the spinal cord was immersed in oxygenated recording solution (ASCF) that consisted of (in mM): NaCl, 125; KCl, 2.5; 1.25 NaH_2_PO_4_ 1.25; CaCl_2_, 2; MgSO_4_, 1.3; HEPES, 10; glucose, 10, pH 7.4. Stimulation of the longitudinal spinal cord sections was performed with a Grass SD9 square pulse stimulator that was connected to both the stimulating electrodes, and controlled by Clampex 10 software, via connection with the 1,550 series digidata. The recorded data was collected using an Axon 200B and signals were filtered with a low-pass 5-kHz filter digitized (Digidata 1550 series) at 32 kHz, and stored and analyzed on a computer using Clampfit 10 software (Molecular Devices, Sunnyvale, CA, USA). Electrical stimulus response relationships were recorded by varying the stimulus intensity from 1 to 100 mA and measuring the amplitude of corresponding evoked compound action potentials (CAPs) that were generated. After electrophysiological recordings, the spinal cord was cut in half at the midway point between the recording and stimulating electrodes. This would result in loss of the signal, but not that of the artifact. Furthermore, tests with tetrodotoxin (1.5 μM) were used to stop generation and propagation of action potentials. These controls determined the extent of artifact in the recordings generated (see **Figure 2**). Data was stored on a PC with windows XP and analyzed using Clampfit 10.5.

### Collection of Electrophysiological Data

The electrically evoked CAPs were analyzed according to their baseline to peak amplitude and conductance velocity. Stimulus response relationships were recorded at 1 mA and measuring the amplitude of corresponding graded CAPs. The latency was measured as the time from stimulus artifact to the peak of CAP, and the conduction velocities were calculated by dividing the distance between the stimulating electrode and the recording electrode (5 cm) by the latency. All analysis of resultant evoked CAPs was performed using Clampfit 10.

### Gait Analysis

Motor and limb function recovery was assessed using video-based gait pattern recording of hindlimb movement (16). The limbs were shaven, and four anatomical landmarks (hip, knee, ankle and toe) were marked on the lateral side of both hindlimbs. These landmarks were identified by palpation by moving the joints. Markers were used to determine the full range of motion (ROM) of the thigh, leg, and foot segments through the stance and swing phase of the gait cycle from toe contact to toe off.

A quantitative video-based gait pattern analysis was adapted from a previous study (16). Briefly, a transparent walking track (rat walk) was built out of Plexiglass (80 cm long, 6 cm wide, 12 cm high). The lateral limb landmarks were recorded using a video camera (Panasonic PV-GS39 MiniDV Camcorder) placed 1 m away from the rat walk. For temporal resolution, the video capture rate was set at 60 frames per second (fps) with a shutter speed of 1/500 s. Three 10 cm markers were equally spaced on top of the rat walk in order to calibrate for distance and velocity.

Prior to video recording, animals were trained to walk across the rat walk. A trial was considered successful when the rats took four consecutive steps without pause. Rats were recorded on week 8 to assess changes in thigh, leg and foot segment angular kinematics (angular position and velocity).

Video recordings were acquired using Vicon Motus (version 9.2) software, and then digitized using MaxTRAQ v. 2.2.0.1. The data was smoothed using a low pass Butterworth filter (cutoff frequency of 10 Hz) in two passes (forward and reverse) to correct for phase distortion using the Signal package for R (version 3.2.3). Sequential lateral images were used to obtain thigh, leg and foot segment kinematics for a minimum of 1 step cycle, and these measures were used to determine the minimum and maximum segment angles (absolute angles with respect to the horizontal axis) to calculate ROM and maximal segment velocities.

### Data Processing and Statistical Analysis

Treatment effects on electrophysiological parameters were estimated using gamma generalized linear mixed models with log link function using the lme4 package (version 1.1-12) for R [version 3.2.3; (17)]. Between-animal variation was modeled as a random intercept term. The null hypotheses of no treatment effects for LAM, ISO, and IbTx were assessed using general linear hypothesis tests, with one-sided *p*-values adjusted for multiple testing, using the multcomp package (version 1.4-5) for R. Tukey box plots were used to show the data, which express the median with inter-quartile rante (IQR) and whiskers depict the extremes of the data or 1.5 × the IQR, whichever is nearest.

A generalized additive model (GAM) was fitted to the angle data. The GAM included parametric fixed effects for Treatment and Body Part, plus their interaction, and a semi-parametric spline term for each combination of treatment and body part to represent how the response variable (angle) changed as a function of the proportion of step completed. The semi-parametric splines can be thought of as population-level effects modeling how the angle of each body part changed through the step-cycle averaged over all animals in each treatment group. Each of these population-level splines is allowed to have a different degree of smoothness.

Individual animal-level deviations from these population-level effects were modeled via a random factor-smooth spline basis. This is the equivalent of a random slope and intercept term in a classic linear mixed effects model, but with the random slope replaced by a random spline. The smoothness penalty for the individual level random factor-smooth splines was based on the first derivative of the spline ensuring the penalty controlled the degree of deviation from the population-level spline for each individual animal (18). The same degree of smoothness (the smoothing parameter) was used for each individual-level deviation spline.

Symbolically, the model fitted is

(1) $$\begin{array}{l} y_{i}=X_{i}\beta +f_{jk}\left(pStep_{i}\right)+f_{l}\left(pStep_{i}\right)+\varepsilon_{i} \end{array}$$

where *X*_i_β is the parametric terms for the model constant, and treatment and body factors plus their interaction. *f*_jk_ indexes the population-level splines for all combinations of the *j*th treatment group and the *k*th body part for the *i*th observation. The subscript *l* denotes individuals, with *f*_*l*_ representing the individual-level random-factor smooth spline. *pStep*_*i*_ is the proportion of the step cycle for the *i*th observation.

The GAM was fitted using the mgcv package (version 1.8.16) for the R statistical language (version 3.3.1 patched). Smoothness selection was performed using residual marginal likelihood (REML), employing the double penalty approach of Marra and Wood (19) to add additional shrinkage to model terms. This has the effect of shrinking the individual-level effects toward the population-level effects.

## Results

### Effect of BK Activation on Tissue Morphology Post-acute SCI

**Figure 1:**
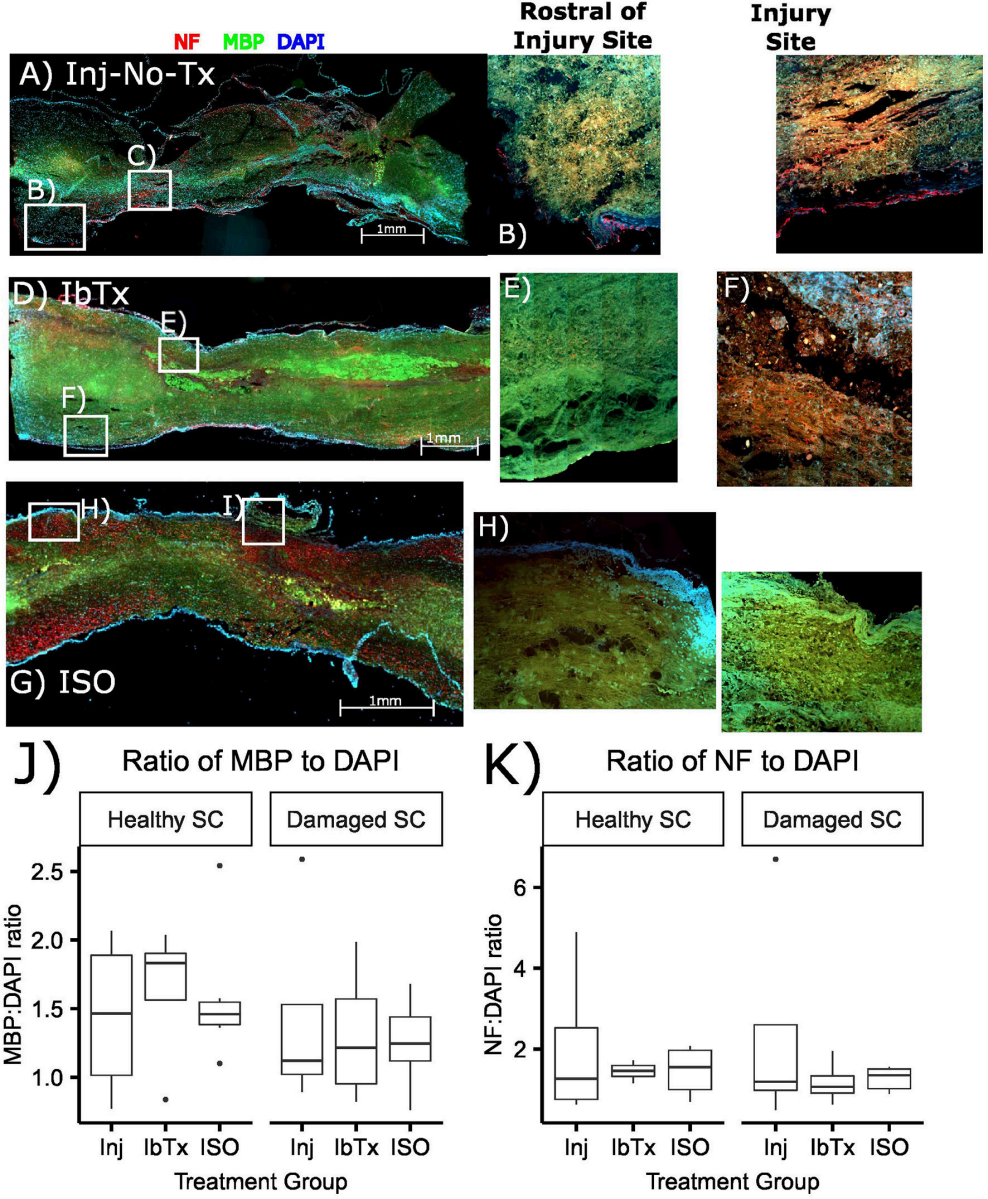
Treatment of SCI with ISO maintains the myelination of axonal tracts. Spinal cords were sectioned and stained with NF (red), MBP (green), and DAPI for nuclear stain (blue) as described in Materials and Methods. **(A–C)** Injured non-treated (Inj-No-Tx); **(D–F)** IbTx treated; **(G–I)** ISO treated; **(B,E,H)** ROIs rostral to the injury site; **(C,F,I)** ROIs of the injury site of the spinal cords. **(J,K)** A semi quantitative comparison between study groups measuring MPB and NF ratios to DAPI.

In order to investigate the extent of myelinated neurons post-SCI, rats underwent a 25 g aneurysm clip injury to the thoracic spinal cord (T5-T7). Drugs were administered 1 h post-injury and rats were then sacrificed at 8 weeks post-injury. Spinal cords were sagittally sectioned and stained with Myelin Basic Protein (MBP- green), and to determine extent of axonal tracts, sections were stained with Neurofilament (NF- red) antibodies. Topographical determination of the localization of these proteins, in conjunction with DAPI, gives an indication of the extent of myelinated neuronal tracts present in healthy and injured tissue in the various treatment groups. Sagittal slices were performed to give a better idea as to the extent of myelination loss in the injured area compared to healthy tissue. Rats that received SCI but no treatment (Inj-No-Tx), had greater loss of myelinated axonal tracts (Figure 1), although this semi-quantitative analysis was not significant (Figure 1J). In our sagittal slices, there was a decrease in MBP on surviving axonal tracts in the injured area in Inj-No-Tx animals compared to areas rostral to the site of injury (Figure 1). Furthermore, the loss of myelination extended beyond the injury site (Figure 1A), likely due to the role of secondary injury mechanisms during SCI. There were more myelinated axons on the rostral, healthy portion of the spinal cord compared to the injured portion (Figures 1B,C,J).

Our working hypothesis is that BK activation leads to greater axonal and glial sparing post-SCI. Thus, we expected treatment with the BK channel blocker IbTx to would cause further damage to the spinal cord. In these specimens we did not see any significant difference between the site of injury and those sites that were rostral to the site of injury (Figures 1D–K), this is similar to that of the Inj-no-Tx group.

In contast to Inj-no-Tx group and IbTx, in ISO-treated animalswe observed more myelin and neuronal markers in the injured area although a semi-quantitative analysis did not reach significance (Figures 1G–J). In contrast to either IbTx or Inj-no-Tx group, there did appear to be less loss of myelin at the site of injury, thus suggesting that there was some morphological sparing occurring at the site of injury in ISO-treated animals.

### ISO-Treated Animals Exhibit Improved Electrophysiological Profile

**Figure 2:**
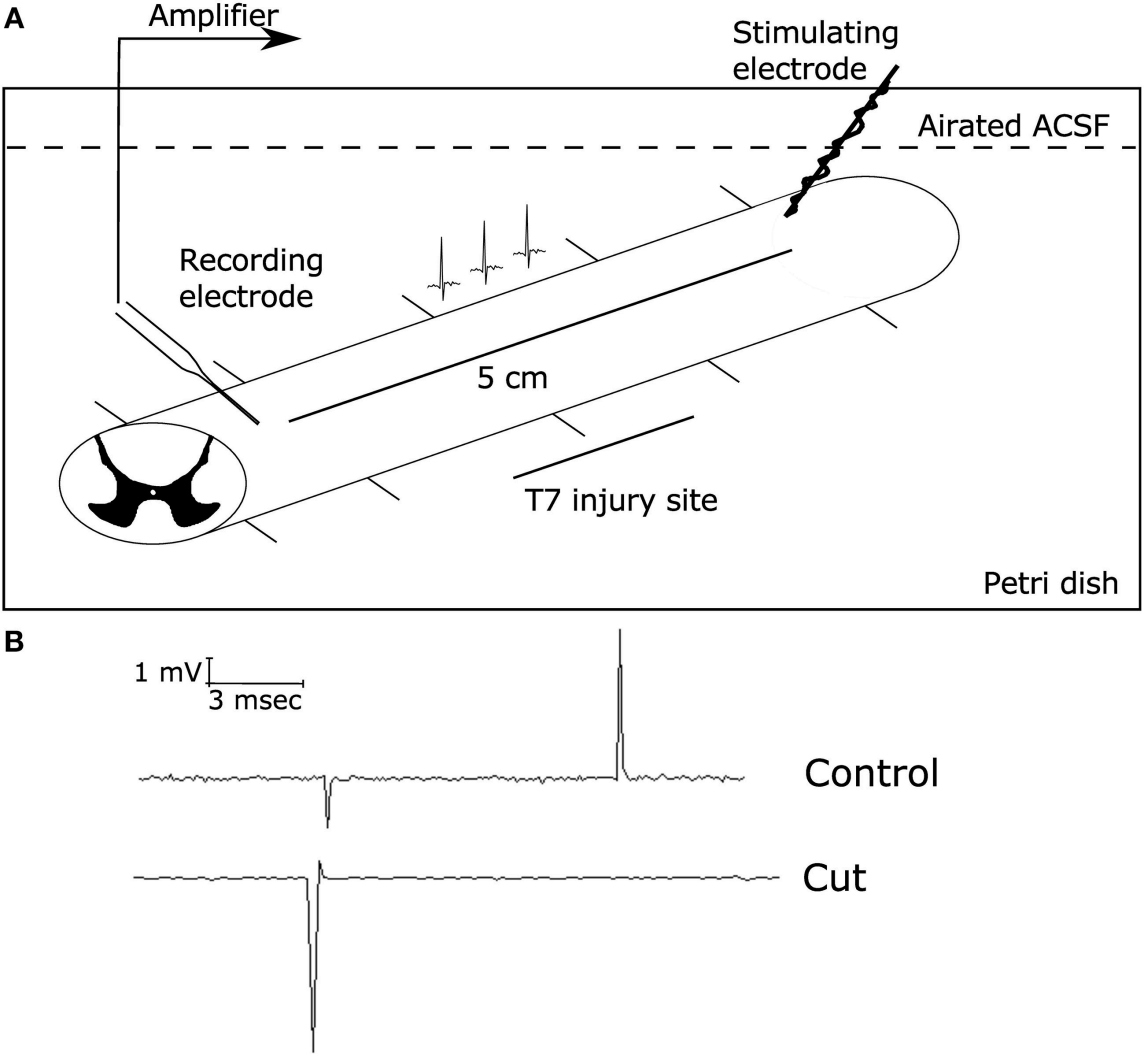
Electrophysiological set-up. Electrophysiological studies were conducted using a suction electrode. **(A)** Diagram demonstrating the electrophysiology set up with the stimulating electrode on the rostral side of the injury (8 weeks post-SCI) and the suction electrode (recording electrode) on the caudal side of the injury site. This allowed for local recordings of the compound action potential propagation through the injury site, as described in Materials and Methods. The initial deflection represents the stimulus artifact, while the second spike is due to the generation of action potentials along axonal tract. **(B)** Representative traces of CAPs on a health spinal cord stimulated at 1 mA for 0.1 ms before (top) and after (bottom) cutting the spinal cord between the two electrodes, demonstrating that the CAP recordings were successful. When the spinal cord was severed, there was no measured CAP present.

Our IHC data suggest that animals that were treated with the BK channel activator ISO, resulted in greater levels of axonal and glial survival post-SCI compared to the Inj-No-Tx group. We next sought to determine whether this increase in cell survival translated into better electrophysiological outcomes in these animals. To characterize the conduction capability of surviving axons in our model, we recorded CAPs from dissected spinal cords (Figure 2 for set-up). A CAP is the “summation” of all the action potentials produced by all the axonal tracts that were stimulated to form an action potential. The spinal cord consists of thousands of axons whose size, myelination status and position with respect to the stimulating and recording electrodes all affect the size of their contribution to the CAP.

With this technique, we can further corroborate whether our IHC findings translated into improved electrophysiolgical function as determined via response to stimulus intensity, CAP amplitude and conduction velocity. After the spinal cord has been injured, animals were treated with ISO or IbTx for 4 weeks, due to the limitation in mini osmotic pump size. Animals were observed for a further 4 weeks. Thus, spinal cords were harvested 8 weeks post-injury. As the administered treatment was only during the acute phase of injury, thus the final long-term effects observed from these isolated spinal cords would be from this initial time period. This is of great interest as we seek to develop pharmacologically relevant treatments for SCI. In generating CAP, we varied the stimulus intensity was from 0.1 to 100 mA. However, to simplify statistical analysis, we used a 1 mA stimulus to compare CAP size between the groups. Thus, we selected the minimal stimulus to elicit a response, which was 1 mA. In our healthy controls (e.g., laminectomized animals), this was the minimal amount of stimulus required to generate a CAP and thus we deemed it to be a healthy standard to compare the other treatment groups to. We found that at lower stimulus intensities, no CAPs were generated. In contrast, higher CAP intensities, we would be able to record only a few CAPs, at which point further recordings were impossible. We assumed that this was the result of damage of the relatively high stimulus intensity.

**Figure 3:**
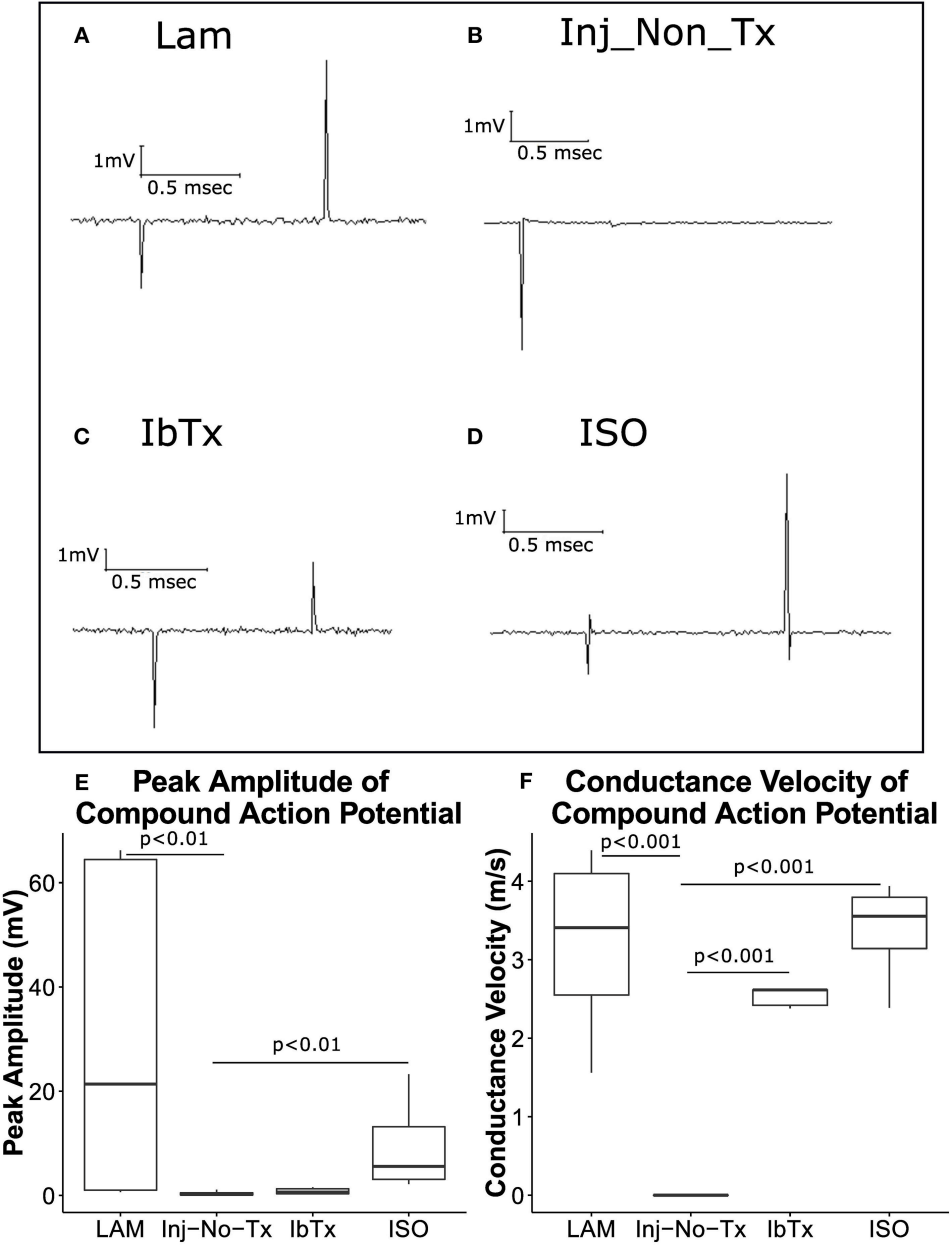
ISO-treated animals exhibit greater axonal survival and CAP velocities compared to injured non-treated animals. Eight weeks post-injury, animals were sacrificed, and spinal cords removed for electrophysiological analysis. Representative traces are shown of CAPs after stimulation of spinal cords at 1 mA for 0.1 ms. **(A)** LAM (laminectomy only); **(B)** injured non-treated (Inj-No-Tx); **(C)** IbTx treated; **(D)** ISO treated. The arrows indicate the artifact upon stimulation of the spinal cord followed by the CAP. Peak Amplitude of CAPs **(E)** and conduction velocity **(F)** were measured. Injured non-treated animals (Inj-No-Tx) exhibited significantly lower peak amplitudes and CAP velocities compared to laminectomised (LAM) animals and animals treated with ISO. Tukey box plots are shown. LAM *n* = 3; Inj = 4; IbTx = 4; ISO = 6.

Compared to laminectomized controls, CAPs obtained from injured non-treated rats (Inj-No-Tx) expressed significantly diminished CAP amplitudes (median 21.36 ± 28.32 mV vs. 0.2 ± 0.49 mV, respectively, Figures 3A,B,E, *p* < 0.01). ISO-treated animals had CAP amplitudes that were comparable to laminectomized controls (median 5.56 ± 7.48 mV, Figures 3D,E), and were significantly higher than spinal cords isolated from Inj-No-Tx animals (*p* < 0.01). Interestingly, there was no significant difference in CAP amplitudes between animals treated with IbTx (median 0.62 ± 0.59 mV, Figures 3C,E) and the Inj-No-Tx group which would suggest that IbTx treatment during acute SCI did not exacerbate the amount of injury in the spinal cord. An alternative explanation is that the maximal amount of injury has already occurred in the spinal cord and any subsequent treatment with inhibitor had not further effect. As stated above, and in line with our overall hypothesis, we found that CAPs from ISO-treated animals were not significantly different from that of laminectomized (health non-injured) spinal cords.

### Surviving Axonal Tracts Exhibit Normal Conduction Velocity

CAP conduction velocity can be used as an indicator of health and thus normal physiology of the studied tissue. Myelinated axonal tracts would be expected to have greater conduction velocity compared to non-myelinated axonal tracts. The distance between the recording electrode and stimulating electrodes were measured for each spinal cord, and using these measurements, CAP conduction velocity was calculated. While CAP velocity was measured over a range of stimuli (0.1–100 mA stimulus), we selected 1 mA of stimulus to compare velocities between the various groups (See above). CAP velocity from laminectomized rats (Figure 3F; median 3.4 ± 1.15 m/s), injured rats treated with IbTx (median 2.6 ± 0.12 m/s), and injured rats treated with ISO (median 3.5 ± 0.6 m/s) were significantly faster than those of Inj-No-Tx animals (0.1 m/s; *p* < 0.001). The median velocities from laminectomized rats and ISO-treated animals had comparable speeds, and were not significantly different (Figure 3F). These findings with those above would suggest that treatment with ISO is neuroprotective and results in improved electrophysiological function post-injury. Unlike our CAP amplitude study, where IbTx-treated animals were not significantly different than the Inj-no-Tx group, the CAP velocity of IbTx-treated animals was significantly different from the Inj-no-Tx group. It could be that while both can elicit CAP, the IbTx-treated group likely has more functional myelinated tracts.

### Improved Neuro-Protection Results in Improved Motor Function in Spinal Cord Injured Rats Treated With ISO

**Figure 4:**
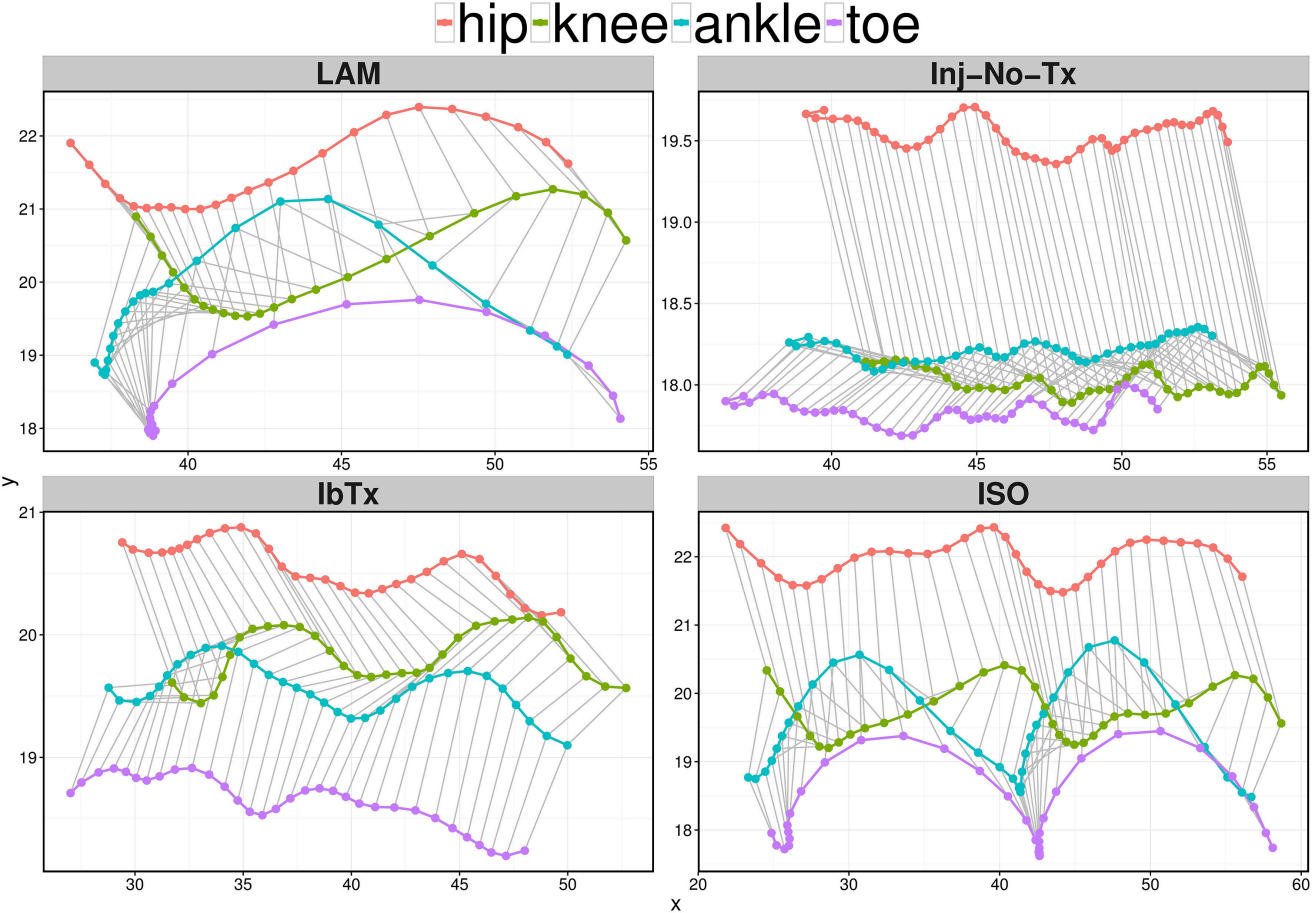
Representative stick figures of angles between the 4 kinematic joints depicting one (LAM) or two (Inj-No-Tx, IbTx, and ISO treated) step cycles. Stick figures of angles between the hip (red), knee (green), ankle (blue), and toe (purple) are depicted of one representative from each treatment group. ISO treated animals demonstrated two clear step cycles, similar to the one step cycle demonstrated from the LAM group. The Inj-No-Tx and IbTx treated representative shows no clear swing phase in the step cycle.

**Figure 5:**
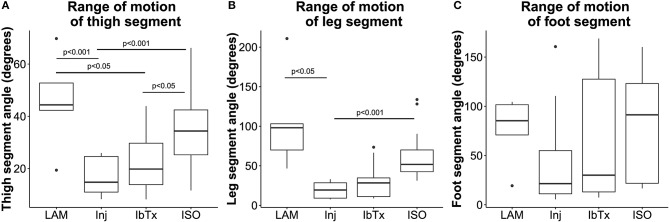
Improved neuro-protection translates to improved ROM in spinal cord injured rats treated with ISO. Eight weeks post-injury, 2D gait analysis was performed as described in Materials and Methods. Maximum thigh **(A)**, leg **(B)**, and foot **(C)** segment angles were assessed. Laminectomised rats and SCI rats treated with ISO had significantly greater ROM of thigh and leg segment angles compared to injured non-treated animals. There were no significant differences in foot segment angle ROM. Tukey box plots are shown. LAM *n* = 3; Inj = 6; IbTx = 6; ISO = 13.

**Figure 6:**
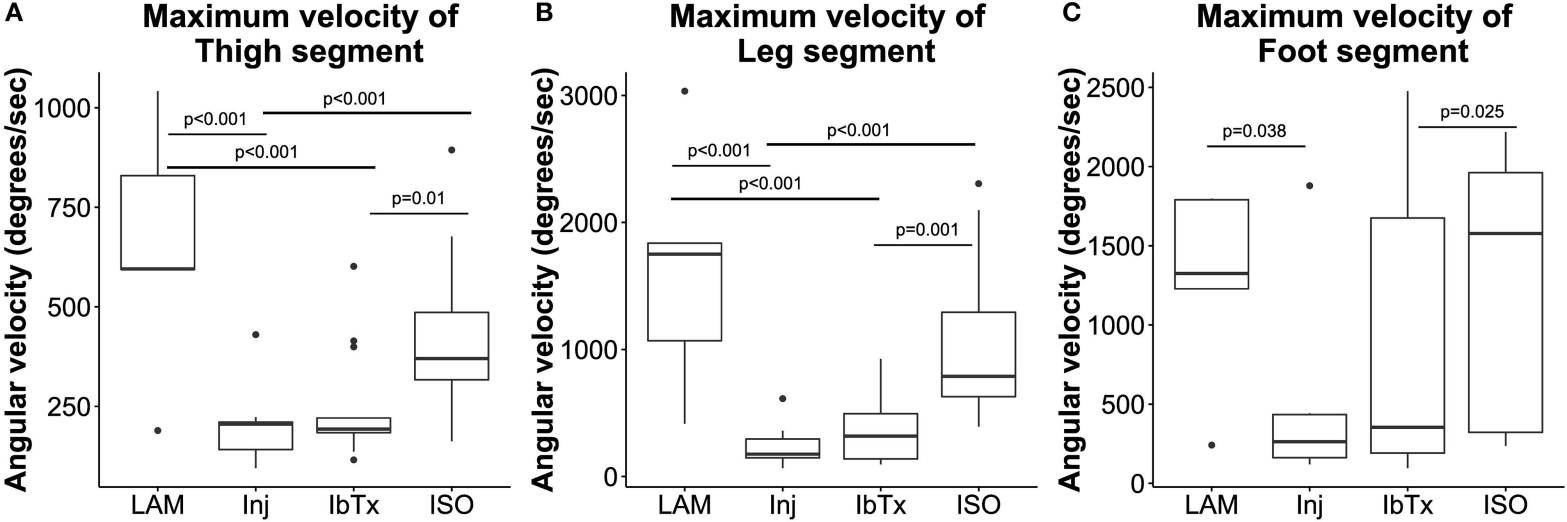
Improved neuro-protection translates to improved motor function in spinal cord injured rats treated with ISO. Eight weeks post-injury, 2D gait analysis was performed as described in Materials and Methods. Maximum velocity of thigh **(A)**, leg **(B)**, and foot **(C)** segment angles were assessed. Laminectomised rats and SCI rats treated with ISO had significantly greater ROM of thigh and leg segment angles compared to injured non-treated animals. There were no significant differences in foot segment angle velocity. Tukey box plots are shown. LAM *n* = 3; Inj = 6; IbTx = 6; ISO = 13.

Our electrophysiological and IHC data suggest that ISO-treated animals experience improved neurological outcomes post-acute SCI. Thus, we wanted to determine whether there were any differences in overall motor function between the different groups. Post SCI, there is a significant decrease in motor function, with deficits in both ROM of limb joints and velocity of limb movement. With SCI impairment, the first loss of function would be at the ankle, with progressive extent of impairment with more proximal aspects of the limb observed with more significant injury. Using a digital camera and computer program, we performed 2D gait analysis starting 1 week post-injury for a duration of 8 weeks. Figure 4 shows one (for Laminectomized animals) or two (for Inj-NT, ISO treated and IbTx treated animals) complete gait cycles 8 weeks post-injury and treatment using stick figures. From the pattern, it is clear that animals treated with ISO had step cycles that looked similar to the laminectomized animals. This data was used to calculate the maximum ROM (Figure 5) and joint segment velocity (Figure 6) of three joint segments (thigh, leg and foot).

Rats that had undergone laminectomy surgery without SCI (Lam) demonstrated the greatest ROM in thigh (median 44.4 ± 18.26°), leg (median 98.1 ± 63°), and foot (median 85.3 ± 34.61°) segment angles (Figures 5A–C), whereas Inj-No-Tx animals demonstrated significantly reduced ROM in thigh (median 14.8 ± 6.84°; *p* < 0.001) and leg (median 19.4 ± 9.77°; *p* < 0.05) segment angles (Figures 5A,B). Whilst the ROM of the foot segment was lower in injured non-treated animals (median 21.4 ± 48.13°) compared to laminectomized rats, this difference was not significant.

We hypothesized that BK activation leads to greater axonal and glial sparing post-SCI, and therefore expected treatment with IbTx, a BK channel inhibitor, to cause further damage to the spinal cord and worsen rat hind limb mobility. However, SCI animals treated with IbTx had median segment angles (thigh median 19.9 ± 11.75°; leg median 28.38 ± 21.35°; and foot median 30.05 ± 61.95°) that were greater than Inj-No-Tx animals, although this increase in ROM was not significant across all segments. In line with our working hypothesis, animals treated with ISO had significantly higher median thigh (median 34.37 ± 12.97°) and leg (median 51.75 ± 28.12°) segment angles (*p* < 0.001) compared to In-No-Tx animals, and higher median thigh segment angle compared to IbTx treated animals (*p* < 0.05). Foot ROM in ISO treated animals (median 91.37 ± 52.2°) was of a similar magnitude to lamenectomised animals, but was not significantly higher than Inj-No-Tx or IbTx treated animals (Figure 5).

Thigh, leg and foot segment velocity was assessed. Lamenectomised animals had significantly greater maximum velocities of all three segments (median maximum velocity of segments: thigh: 595.37 ± 317.82°/s; leg: 1750.8 ± 976.56°/s; foot: 1325.23 ± 634.89°/s) compared to Inj-no-Tx animals (thigh: 205.53 ± 86.93°/s; leg: 176.28 ± 149.61°/s; foot: 262.83 ± 481.25°/s; *p* < 0.001 in thigh and leg segment velocity, p < 0.05 for foot segment velocity, Figures 6A–C). There was no significant difference in median segment velocities of IbTx treated animals (thigh: 192.95 ± 140.13°/s; leg: 317.77 ± 241.21°/s; foot: 354.03 ± 843.58°/s) compared to Inj-no-Tx. Thigh and leg segment velocities, in IbTx-treated animals were significantly slower than in lamenectomised animals (*p* < 0.001, Figures 6A–C). ISO treated animals had significantly faster velocities in their thigh (median 370.1 ± 172.68°/s) and leg (median 789 ± 578.1°/s) segments compared to Inj-No-Tx animals (*p* < 0.001) and IbTx treated animals (*p* = 0.01 and *p* = 0.001, respectively; Figures 6A,B). ISO treated animals had significantly higher foot segment velocities (median 1577.52 ± 790.93°/s) than IbTx treated animals (*p* < 0.05, Figure 6C).

**Figure 7:**
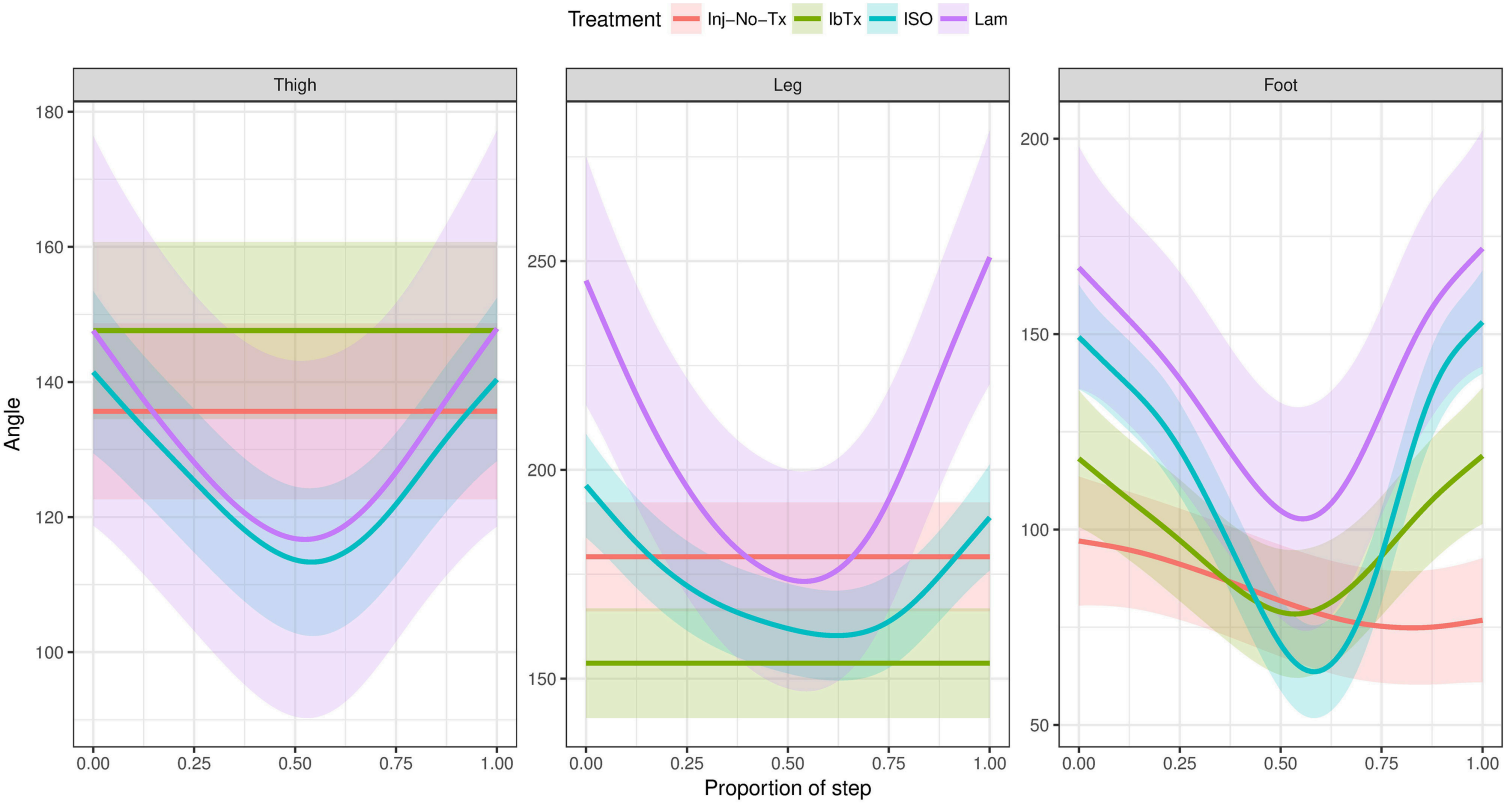
Generalized Additive Mixed Model of joint segment angles through a step cycle. The 2D gait analysis was used to calculate angles of the thigh, leg and foot segments through the proportion of the step cycle, and modeled using a GAM. Inj-No-Tx (red) and IbTx treated (green) animals demonstrated no swing phase in gait cycle in thigh and leg segment angles. Foot segment angles in the Inj-No-Tx and IbTx treated animals demonstrated some range of movement, although the mobility was less than in LAM (purple) and ISO (blue) treated animals. ISO treated animals demonstrated a classic swing phase in thigh, leg, and foot segment angles through the proportion of the step cycle, mirroring the LAM animals.

Finally, change in joint segment angles was modeled through the proportion of the step cycle using a Generalized Additive Mixed Model (Figure 7). Lamenectomised animals demonstrated a classic swing phase through the proportion of the step cycle in all three segment joints measured. Furthermore, the change in segment angles from ISO treated animals mirrored the lamenectomised control animals, demonstrating that these animals were able to walk as efficiently as lamenectomised animals. On the other hand, Inj-No-Tx animals as well as IbTx treated animals had no change in thigh and leg segment angles throughout the step cycle, but did demonstrate some change in the foot segment angle, although the range of movement was limited.

These data demonstrate that 8 weeks post-SCI, acute treatment with ISO improved motor function, allowing these rats to walk, compared to their non-treated counterparts, who had limited or no use of their hind limbs.

## Discussion

Here we have demonstrated, for the first time, the neuroprotective properties of BK channel activation in acute SCI. Treatment of acute SCI with ISO led to an increase in myelinated axonal tracts as well as improved electrophysiological and motor function. We have previously demonstrated that BK channels are exposed in a chronic model of SCI, and this exposure exacerbates the motor deficits experience post-SCI (13). In the previous study we determined that BK channels are indeed expressed in the spinal cord and that they contribute to the altered electrophysiology of the chronically injured spinal cord. This previous study only examined the expression of the BK channel and did not examine the role of BK channel activity during the acute phase of SCI injury. To further this previous study, here we examined whether targeting the BK channel in the acutely injured spinal cord would result in greater axonal and glial survival, and thereby improve neural and motor function. An advantage of targeting the BK channel is that it is located on both glia and neurons. In targeting this ion channel, one is able to potentially induce sparing of both cell types, unlike in other pharmacological treatments, where only one cell type is spared (18, 20).

Our exciting present findings do demonstrate that BK channel activation is indeed neuro-protective during acute SCI. However, it is unclear if the myelination observed was due to neuroprotection, regeneration of neurons, or repopulation of neurons from available stem cells. It has previously been demonstrated that endogenous oligodendrogenesis and remyelination occurs spontaneously after SCI (18), although levels of remyelination never recovered to normal levels in our study. The ratio of axonal diameter to myelin is reduced post-SCI (5) and is less than optimal for axonal conduction velocity (21, 22). Furthermore, the diameter of injured axons has been shown to decrease post-injury (5, 23), leading to reduced conduction velocity (23–25).

In our IHC studies, we found that there was a decrease in myelinated axonal tracts in the injured areas of all groups, in comparison to the non-injured areas both rostral and caudal of the injury site using semi quantitative analysis. Although there was a trend for a decrease, this was not significant from other groups at the confidence level that we selected (0.05). However, the ISO treated animals appeared to have more myelinated tracts in the injured site compared to the injury site of the Inj-No-Tx group. It is possible that a greater sample number is required to determine the extent of difference between the groups. Using electrophysiology, we were better able to identify physiological differences between the study groups. CAP will identify differences in extent of axonal myelination (CAP velocity) as well as extent of neuronal injury (CAP amplitude). CAP amplitude was, as expected, higher in ISO-treated animals compared to Inj-No-Tx animals and IbTx-treated animals. The CAP amplitude data suggests that there are significantly fewer functional axons in IbTx-treated animals but not as few as Inj-No-Tx group. The tracts that are present in animal treated with IbTx appear to be functional however. When comparing IHC axonal myelination, the IbTx treated animals had more myelinated tracts in the damaged and rostral portion of the spinal cord compared to injured controls, thus supporting our electrophysiological findings. It is unclear if this is due to tissue sparing or an increase in tissue repair. We would have expected that animals treated with BK channel inhibition, via IbTx, should have caused an increase in neuronal degradation, increased impairment of motor function and a worse electrophysiological profile than the Inj-non-Tx animal group. The reason for this finding could be that maximal degradation in neuronal and glial function is already experienced in both IbTx and Inj-non-Tx groups. An alternative possibility is that the IbTx may have other effects that start to affect the spinal cord during the chronic period of injury. As part of our future studies, we will be investigating the effects of BK channel modulation during the chronically injured spinal cord.

The model of the gait parameters demonstrated that the conduction velocity of ISO treated animals was significantly better than Inj-no-Tx animal group, further supporting our hypothesis. Interestingly, while IbTx treated animals did not improve movement of the thigh and leg segments, there was improvement of the foot segment compared to the Inj-No-Tx group, which was unexpected. While there are different possibilities for this, one hypothesis is that IbTx causes alterations in neural networks in the spinal cord, leading to more distal improvements in motor function. An alternate hypothesis is that while ISO may have beneficial effects early on during the acute phase of injury (first 2 weeks post-injury), treatment with IbTx may start to have further beneficial effects during the chronic phase of injury (at 3 weeks plus). In this study, animals were treated with IbTx for a period of 4 weeks. It may be that the make-up of the BK channel on the spinal cords is either altered by the presence of IbTx, or that somehow the BK channel becomes irreversibly blocked. However, it remains unclear how IbTx may potentially lead to improved motor function compared to injured non-treated animals, even though the improvement was only observed at the most distal segment of the limb. Our treatment paradigm only treated the acutely injured spinal cord for 4 weeks, our future studies will examine these effects during the chronic phase of injury (administering compounds >4 weeks post-injury).

Our findings support our hypothesis that activation of the BK channel with isopimaric acid is neuroprotective during acute SCI. Our model is that during acute SCI and increase in ROS production results in the inhibition of the BK channel. This leads to significant excitotoxicity and tissue loss. By treating animals with ISO, the inhibition of the BK channels via ROS, is reduced. Thus, ameliorating the exocitotoxic effects of ROS. ROS levels are thought to affect the C-terminal region of BK channels, affecting their sensitivity to Ca^2+^ (26). ISO is thought to have no effect on BK channel conductance, but increases the channel's sensitivity to both voltage and Ca^2+^, thus reducing the activation potential of this ion channel (27). We have also previously demonstrated that ROS are able to modulate BK channel activity (28). Others have demonstrated that activation of the BK channel by other activators leads to neuroprotection associated with reduced levels of ROS (29, 30), and may even protect antioxidant enzymes (29) in neural cells, as well as in cardiac mitochondria (31). While IbTx does not significantly improve the parameters we have tested, it does not make them worse compared to Inj-no-Tx group. This is surprising on its own as we would have expected to have some effect. However, there are several possibilities to explain this finding.

First, it could be that the injury environment is already at its maximum and thus treatment with IbTx could not make it any worse. Another possibility is that the ROS producing environment, already maximized BK channel inhibition. Thus, further inhibition via IbTx elicits no further effect. Lastly, it is possible that the inhibition of the BK channel may also have other neuromodulatory effects on the motor neural networks. This latter possibility would be supported by one of our previous studies that suggest endogenous neural stem cells can be modulated via BK channels that are expressed on their surface.

In conclusion, we have shown that targeting of the BK channel in acute SCI leads to improved neural function and more importantly, improved motor function. While the mechanism remains unclear, our current model is that BK channel activation leads to reduced Ca^2+^ influx and thus reduced cell death during SCI. Our future directions are to further investigate this model and to further expand into determining the effects of the BK channel during chronic SCI.

## Ethics Statement

The study was reviewed and approved by the President's Committee on Animal Care, which follows the guidelines and is reviewed by the Canadian Council on Animal Care.

## Author Contributions

JMB was responsible for the generation of the project idea, design of the study, analysis of the results, and generation of the manuscript. JB was responsible for the design of the gait analysis, motion capture system and data acquirement. GS was responsible for the generation of the statistical models to analyze the results throughout the manuscript and gait analysis of animal movement. MJ and KL were responsible for the immunohistochemistry, electrophysiology and gait experiments, including analysis.

### Conflict of Interest Statement

The authors declare that the research was conducted in the absence of any commercial or financial relationships that could be construed as a potential conflict of interest.
